# Hospital and Poor Law Problems

**Published:** 1907-02-09

**Authors:** 


### HOSPITAL AND POOR LAW PROBLEMS.

### THE TREATMENT OF POOR-LAW CASES IN VOLUNTARY HOSPITALS.

In the recent discussions which have taken place on the excessive amount of free medical relief given by the voluntary hospitals, a strong point has been made of the abuse represented by the large number of Poor-law cases admitted to treatment, which could properly be dealt with in the Poor-law infirmaries. Poor-law guardians have the power to grant subscriptions in aid of institutions or objects which tend to diminish the charge upon the poor-law funds. Hospitals come under this category, and boards of guardians frequently make an annual subscription to the hospitals situated in the area over which they exercise jurisdiction. The inadequacy of these subscriptions was brought out at a meeting last Friday, the 1st instant, at the Medical College of the London Hospital. The object of the meeting was to try and induce the guardians in the poor-law unions from which patients were sent to the London Hospital to take a more active interest in the work of this great charity by making their contributions bear some proportion to the cost entailed upon the hospital by the reception of Poor-law cases. At the present time, as the Chairman of the London Hospital pointed out, the guardians gave very meagre subscriptions to the London Hospital. The subscription from the Guardians of the City of London and that from Poplar, for instance, was £21 per annum; the Guardians of West Ham contributed 25 guineas, and those of Hackney 12 guineas; but none of the other guardians gave anything to the London Hospital. How totally inadequate these amounts are is proved by the following facts stated by Mr. Holland. Ten years ago, *i.e.*, in 1896, the expenditure of the London Hospital was £64,000 a year; now it is over £90,000. In the 10 years nearly half a million had been spent on rebuilding the old hospital which had been doing good service for 140 years. The increase in the volume of work undertaken by the London Hospital is shown by the number of patients treated. In 1896 the in-patients totalled 11,300; in 1906 they had increased to 14,139—an increase of 2,839. In 1896 the out-patients amounted to 158,000, and in 1906 they were over 230,000, an increase of upwards of 72,000. The Chairman claimed that the increase in the out-patients was due to the establishment of new departments for ophthalmic cases, nose and ear, lupus, and the "new opsonic work, by which they hoped to conquer that dread disease consumption." Of the total expenditure only £20,000 a year was derived from the rents of the hospital's property adjacent to the institution. Another good point made by the Chairman was that as a matter of mere humanity, and in the interests of the patients, it was desirable to have an understanding between each hospital and each board of guardians in order to secure that, when a poor-law case who was the inmate of a hospital had to be removed to the infirmary, arrangements should be made to transfer such patients direct to their own parishes. The Chairman submitted a list of the number of patients the London Hospital were treating from the various parishes. In making his calculations he had taken the cost of each in-patient at £5 18s. per annum, and on that basis had arrived at the following figures :—

In-patients at the London Hospital, Whitechapel, in 1906.

| Name of Parish. | No. of In-patients. | Cost to the Hospital. £ |
|---|---|---|
| Whitechapel | 1,154 | 5,866 |
| St. George's | 1,019 | 5,180 |
| Stepney | 877 | 4,456 |
| Mile End | 855 | 4,346 |
| Bow | 782 | 3,975 |
| Hackney | 640 | 3,253 |
| Poplar | 545 | 2,770 |
| Stratford | 411 | 2,090 |
| Limehouse | 312 | 1,586 |
| City of London | 191 | 970 |
| Stoke Newington | 81 | 411 |
| Totals | 8,118 | £41,262 |

Out-patients in 1906.

The figures for out-patients (reckoned at 2s. each) were :—

| | | £ |
|---|---|---|
| Bethnal Green | 10,292 | 1,029 |
| Stepney | 10,132 | 1,013 |
| Mile End | 8,076 | 807 |
| St. George's | 6,906 | 690 |
| Bow | 5,100 | 510 |
| Spitalfields | 4,866 | 486 |
| Whitechapel | 4,596 | 459 |
| Hackney | 4,240 | 424 |
| Poplar | 3,639 | 363 |
| Shoreditch | 591 | 59 |
| Totals | 58,438 | £5,840 |

It does not appear to what extent, if at all, the number of patients from the various parishes enumerated in the above tables were sent in by the Guardians or in any sense as Poor-law cases. No evidence was adduced as to the circumstances of the patients, or to show whether any, and if so how many, of them were in receipt of parish relief. It was not made clear whether the 8,118 in-patients and the 58,438 out-patients included in the tables had, or had not, any connection with the work of the Guardians, or any relation to it. Information on these points is essential for the purpose of deciding the extent to which the London Hospital may properly look to Guardians for compensation for treating these patients within its walls. If it is claimed that except for the existence of the London Hospital the whole of these cases would have been treated in the Poor-law infirmaries, then it is clear that more than half the annual expenditure upon patients at the London Hospital might, under a better system, be saved, because in such a case it would properly devolve upon the Guardians and not upon the hospital authorities. No addition to the annual subscriptions of the Guardians is likely to amount to a sum which can approach £47,000 per annum, the actual expenditure annually involved, as the above tables show.

### Several Points Raised.

The meeting cannot fail to be useful because it has called attention to precise figures, which, on the face of them, should yield instructive information, if they are thoroughly and exhaustively treated, so that all the facts may be clearly demonstrated in regard to the actual proportion of this £47,000 a year, spent by the London Hospital, which ought, properly, to be borne by the Poor-law authorities. One of the Guardians present complained that the London Hospital often returned surgical cases to the parish too quickly, that is, before the patient had completely recovered. The chairman replied, that the charge that the London Hospital took cases from the Poor-law and turned them out too soon, could only be answered by pointing out the great difficulty there was, owing to the pressure on its beds, which prevented the hospital from permitting them to be occupied by patients for the purposes of convalescence. The chairman agreed that often in-patients ought to remain longer than they did in the hospital, but what was the hospital to do to prevent it? It was a choice of evils, and the hospital had to turn out cases in order to make room for new patients, whose cases were even more pressing. Replying to a guardian who urged that the number of cases admitted should be limited, as at present many of them either ought to pay for themselves, or to go into the workhouse infirmary, the chairman claimed that no one was admitted to the London Hospital who could afford to pay for a doctor, and that, as to the workhouse infirmary cases, he had called that conference to avoid the necessity of too strict a reference to the Poor-law, which, he thought they were all agreed, was undesirable. The chairman further suggested that, the Guardians having ascertained the amount, which the hospital saved each board by admitting Poor-law cases, should then agree to contribute one-twenty-fourth of the total expenditure each year to the London Hospital. In the result, the meeting agreed that there was no need for delay in the transfer of Poor-law patients from the London Hospital to the infirmaries and workhouses, and that arrangements should be made with the relieving officers to prevent any such delay in future. It was further understood that the various points raised would be brought before each Board of Guardians represented at the meeting.

### A New System Essential.

We shall watch the outcome of this meeting of February 1 with genuine interest.

### The Voluntary Hospitals and Poor-law Guardians.

The present relations between the voluntary hospitals and the Poor-law Guardians are anomalous. On the one hand, accident cases are admitted to the Poor-la winfirmaries and payments are exacted, wherever possible, from the patients' friends by the Guardians, though such cases are treated without payment at the voluntary hospitals. On the other hand, as the figures quoted at the meeting indicate, a large sum of money is expended at the present time by some or all of the voluntary hospitals upon Poor-law cases, though in fact the money is raised for the benefit of the sick poor who are not provided for under the Poor-law, and who are above the pauper class, being, in fact, self-respecting and self-supporting citizens. These facts illustrate the anomalies of the present relations between voluntary hospitals and Poor-law authorities. The whole position in regard to these matters requires reconsideration and readjustment. Under a reformed system the Poor-law, with its modern and well-equipped infirmaries, might relieve the voluntary hospitals of much of the work which they at present do, though it does not properly belong to them, and the Poor-law infirmaries might and ought to be made available for the training of medical students. If these question were to be intelligently dealt with, the position of London, as the centre of medical education, would be immeasurably strengthened; the voluntary hospitals would be provided with adequate funds to do all the work which properly devolves upon them; the Poor-law infirmaries would take their proper place in the existing system of medical relief, and every poor person, when ill, would then secure the maximum of efficient treatment with the minimum of difficulty and trouble to themselves and their families.

